# Superfluorinated Extracellular Vesicles for In Vivo Imaging by ^19^F-MRI

**DOI:** 10.1021/acsami.2c20566

**Published:** 2023-02-13

**Authors:** María Sancho-Albero, Nazeeha Ayaz, Victor Sebastian, Cristina Chirizzi, Miguel Encinas-Gimenez, Giulia Neri, Linda Chaabane, Lluís Luján, Pilar Martin-Duque, Pierangelo Metrangolo, Jesús Santamaría, Francesca Baldelli Bombelli

**Affiliations:** † Instituto de Nanociencia y Materiales de Aragón (INMA), CSIC-Universidad de Zaragoza, 50009 Zaragoza, Spain; ‡ Department of Chemical Engineering and Environmental Technologies, University of Zaragoza, 50009 Zaragoza, Spain; § Networking Research Center on Bioengineering Biomaterials and Nanomedicine (CIBER-BBN), 28029 Madrid, Spain; ∥ Laboratory of Supramolecular and Bio-Nanomaterials (SupraBioNano Lab), Department of Chemistry, Materials and Chemical Engineering, “Giulio Natta”, Politecnico di Milano, 20131 Milan, Italy; ⊥ Experimental Neurology (INSPE) and Experimental Imaging Center (CIS), Neuroscience Division, IRCCS Ospedale San Raffaele, 20132 Milan, Italy; # Department of Animal Pathology, University of Zaragoza, 50009 Zaragoza, Spain; ∇ Instituto Universitario de Investigación Mixto Agroalimentario de Aragón (IA2), University of Zaragoza, 50009 Zaragoza, Spain; ○ Instituto Aragonés de Ciencias de la Salud (IACS) /IIS Aragón, Zaragoza 5009, Spain; ◆ Fundación Araid, 50018 Zaragoza, Spain

**Keywords:** bioimaging, PERFECTA, ^19^F-MRI, fluorine, extracellular vesicles

## Abstract

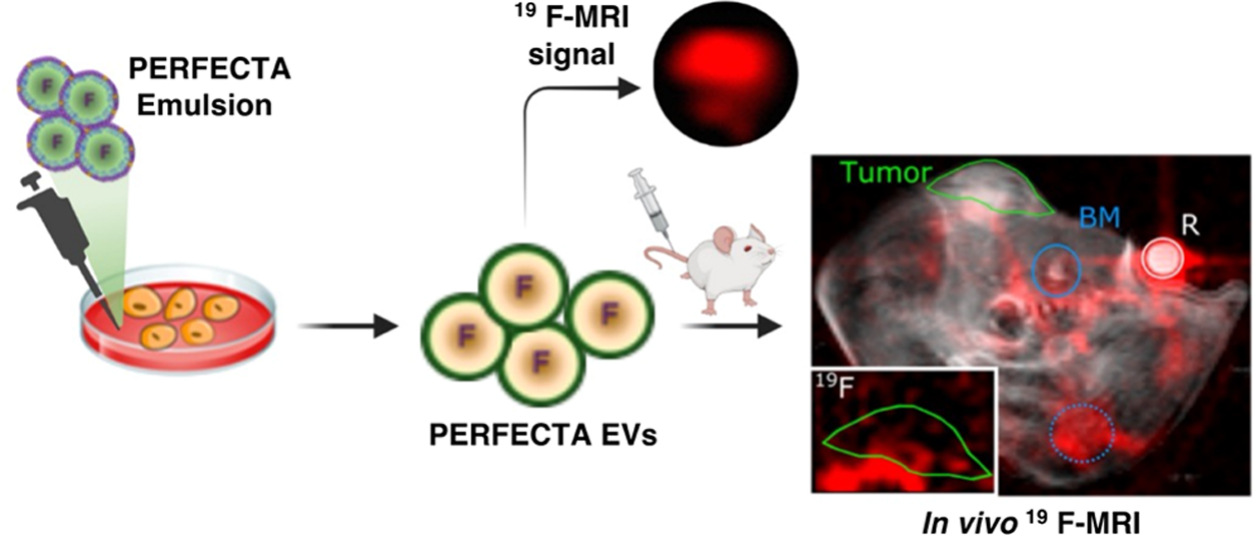

Extracellular vesicles (EVs) play a crucial role in cell-to-cell communication and have great potential as efficient delivery vectors. However, a better understanding of EV *in vivo* behavior is hampered by the limitations of current imaging tools. In addition, chemical labels present the risk of altering the EV membrane features and, thus, in vivo behavior. ^19^F-MRI is a safe bioimaging technique providing selective images of exogenous probes. Here, we present the first example of fluorinated EVs containing PERFECTA, a branched molecule with 36 magnetically equivalent ^19^F atoms. A PERFECTA emulsion is given to the cells, and PERFECTA-containing EVs are naturally produced. PERFECTA-EVs maintain the physicochemical features, morphology, and biological fingerprint as native EVs but exhibit an intense ^19^F-NMR signal and excellent ^19^F relaxation times. In vivo ^19^F-MRI and tumor-targeting capabilities of stem cell-derived PERFECTA-EVs are also proved. We propose PERFECTA-EVs as promising biohybrids for imaging biodistribution and delivery of EVs throughout the body.

## Introduction

Extracellular vesicles (EVs) are secreted by almost all cell lines,^1^ with key roles that include cellular signaling to close and distal cells by the transport of active biomolecules such as lipids, proteins, or nucleic acids.^2,3^ In addition, the inherent properties of EVs such as abundance in body fluids, high stability, low immunogenicity, targeting capabilities, and ability to cross physiological barriers fueled their use as potential selective vectors capable of delivering active biomolecules and therapeutic nanoparticles (NPs) to specific cells and tissues.^4−7^ However, the translation of EV-based diagnostic and therapeutic approaches to the clinics is strongly hampered by the lack of precise imaging methodologies that allow monitoring EV biodistribution.^8,9^

To date, several imaging methodologies have been used for assessing the in vivo fate of EVs, requiring the addition of probes for their labeling.^10,11^ Optical and nuclear imaging are among the most employed in vivo technologies for EV imaging.^12,13^ While optical imaging protocols are versatile and widely used to study EVs in vitro and in vivo,^11,14^ they do not allow easy quantification and, most importantly, have limited tissue penetration, hampering their clinical translation. In this regard, magnetic resonance imaging (MRI) is particularly attractive as it is characterized by high spatial resolution and rapid acquisition without the use of harmful ionizing radiations or radioactive nuclides.^12^ Conventional ^1^H-MRI can provide anatomical and functional information to imaged EVs using tailored contrast agents.^15^ In this respect, some works proposed the combination of metal-based contrast agents (i.e., Gd-lipids) or superparamagnetic iron oxide NPs (SPIONs), serving as MRI contrast agents, with EVs detected as signal enhancement related to target tissues.^16,17^ The use of SPIONs as contrast agents for tracking EVs by MRI faces the challenges associated with conventional MRI such as low sensitivity and specificity.^16−20^ Also, even if Gd-complexes are the most common MRI contrast agents, their use has lately been related to the accumulation of toxic Gd^3+^ in the brain.^21^ Additionally, the manipulation needed to generate EV-SPION or EV-Gd hybrids (e.g., electroporation to introduce SPIONs/Gd-complexes into EVs or covalent functionalization of the EV external surface to SPION grafting) may produce unwanted modifications in their membrane and compromise their targeting capabilities, a problem that is shared with other labeling methods such as the inclusion of nanoparticles or the chemical grafting of fluorescent tags. Importantly, hot-spot imaging by ^19^F-MRI demonstrated several advantages,^22^ as endogenous fluorine content in the body is below MRI detection limit and thus exogenous fluorinated probes can be imaged with high selectivity on the anatomical images obtained by ^1^H-MRI without a background signal.^23−25^

**Figure 1:**
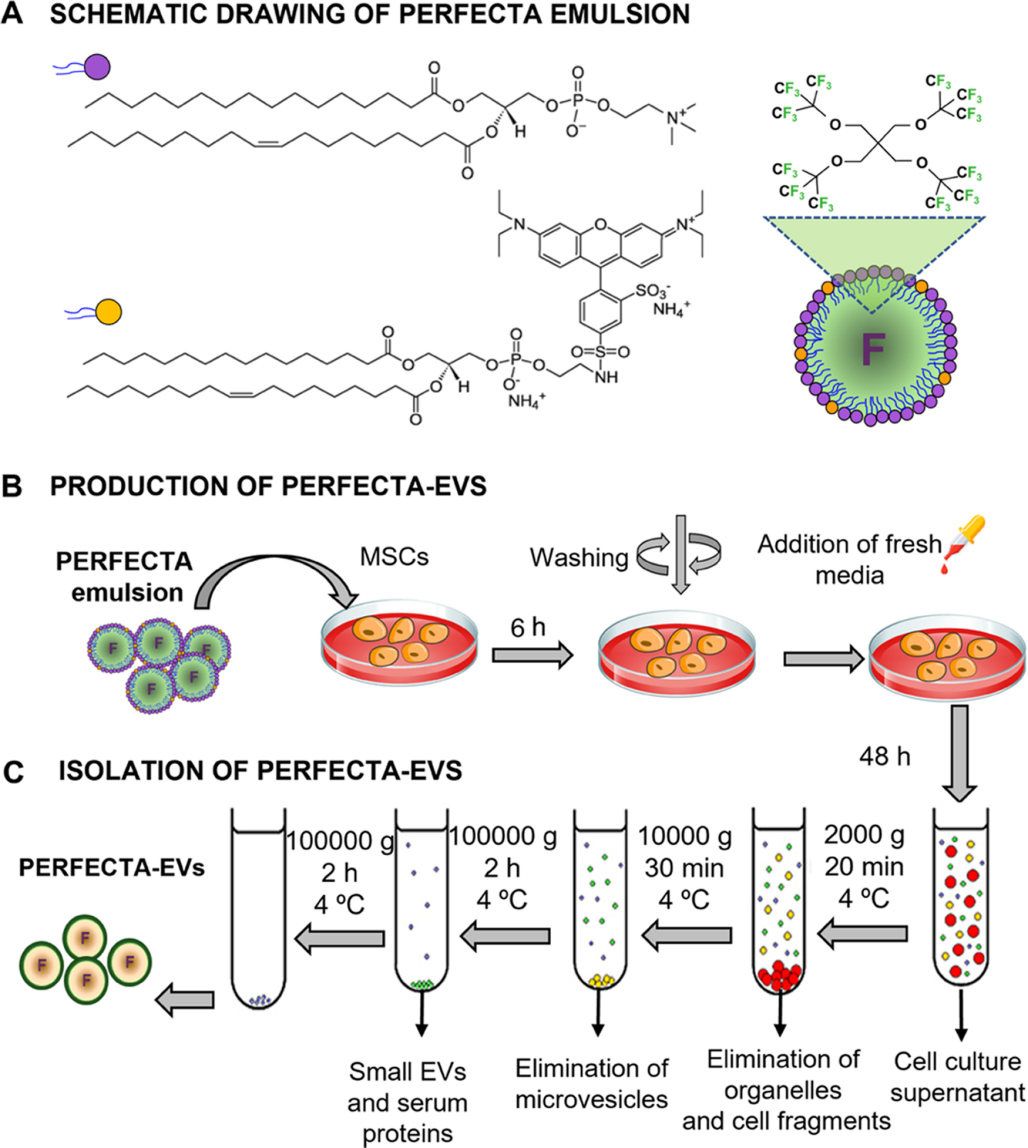
Scheme of the composition of PERFECTA emulsion and production of PERFECTA-EVs. (A) Molecular structure of PERFECTA and main lipid components of the emulsion, and schematic drawing of a fluorescent emulsion droplet. (B) Scheme representing the labeling strategy for incubating parental MSC cells with PERFECTA emulsion and (C) centrifugation protocol to isolate PERFECTA-EVs.

In this work, we designed and developed a simple and efficient strategy that exploits the natural EV biogenesis pathway to produce superfluorinated EVs, enabling their in vivo detection by ^19^F-MRI. Among different ^19^F-MRI probes, we chose PERFECTA,^26−29^ which is a biocompatible highly symmetric branched molecule with 36 magnetically equivalent ^19^F atoms offering excellent imaging properties. It has been previously hypothesized that ^19^F-MRI probes used for cell labeling could be secreted through EVs,^30^ but no experimental evidence has ever been shown. Here for the first time, superfluorinated EVs have been obtained and isolated as imaging biotools. To this end, PERFECTA-EVs were produced incubating the parental cells with a biocompatible lipid emulsion of PERFECTA (Figure 1). In this way, cells internalize PERFECTA as emulsion nanodroplets and expel it again, incorporated in EVs as a PERFECTA-EV system.

## Experimental Section

### Cell Culture

Bone marrow murine mesenchymal stem cells (MSCs) from the C57BL/6 strain were used (Gibco, Thermo Fisher Scientific) and grown in MesenCult basal media (StemCell Technologies Inc.) containing 10% of mesenchymal stem cell stimulatory supplements (StemCell Technologies Inc.), 100 units/mL of penicillin, and 100 g/mL of streptomycin at 37 °C and 5% CO_2_ and 3% O_2_ and were cultured in Dulbecco’s modified Eagle’s F-12 medium (DMEM F12, Gibco) with 10% fetal bovine serum (FBS, Gibco), 1% penicillin/streptomycin, and 1% amphotericin and atmosphere under hypoxic conditions (3% O_2_). For culturing B16-F10 cells (obtained from Cancer Research-U.K. cell services), DMEM with 10% of FBS (GIBCO) supplemented with 1% penicillin/streptomycin and 1% amphotericin (Biowest, France) was used. Furthermore, these metastatic cells were maintained under normoxic conditions. To obtain cell culture media free of EVs (ULTRACEN media), cell media was enriched with 10% of FBS free of EVs (depleted from serum by ultracentrifugation at 100,000*g* for 8 h at 4 °C).

### Synthesis and Characterization of PERFECTA Lipid Emulsion

PERFECTA was synthesized as reported previously.^26^ The lipid emulsion of PERFECTA was prepared, slightly modifying the protocol adopted from previous studies.^27^ Briefly, a fluorescent PERFECTA emulsion was prepared incorporating 0.1% (w/w) of fluorescent lipid (l-α-phosphatidylethanolamine-*N*-(lissamine rhodamine B sulfonyl)(ammonium salt), Avanti Polar Lipids) in an aqueous solution of 4% (w/w) egg lecithin and 4% (w/v) safflower oil. This mixture was added to melted PERFECTA followed by several heat and sonication cycles.^26^ The morphology and size distribution of PERFECTA emulsion were evaluated by transmission electron microscopy (TEM) and dynamic light scattering (DLS). TEM analysis was performed in a T20-FEI Tecnai thermionic transmission electron microscope operated at 200 kV with a LaB6 electron source fitted with a “SuperTwin”. The TEM sample was negatively stained following a previous protocol.^31^ DLS measurements were performed on an ALV apparatus equipped with an ALV- 5000/EPP Correlator, a special optical fiber detector, and an ALV/CGS-3 Compact goniometer. The light source is a He–Ne laser (λ = 633 nm) and a 22 mW output power. Data analysis has been performed according to standard procedures, and autocorrelation functions were analyzed through a constrained regularization method (Laplace inversion of the time autocorrelation functions), CONTIN, for obtaining particle size distribution from which we extracted the mean hydrodynamic radii. ^19^F-NMR measurements were recorded at 305 K on a Bruker AV400 spectrometer operating at 400 MHz for the ^19^F nucleus. PERFECTA emulsion was diluted in a ratio of 1:10 in MilliQ before carrying out the measurements. TFA dissolved in D_2_O was used as an external standard for all NMR measurements.

### PERFECTA Emulsion Cellular Uptake Studies

PERFECTA uptake was performed incubating cell cultures with PERFECTA emulsion at 37 °C. PERFECTA cellular cytotoxicity was evaluated incubating both MSCs and B16-F10 cells with PERFECTA emulsion (at 2, 1, 0.5, 0.25, 0.125, 0.06, 0.03, and 0.015 mg/mL expressed in PERFECTA concentration) for 24 and 48 h, using Blue Cell Viability assay as previously reported.^32^ Cellular uptake of PERFECTA emulsion in MSCs and B16-F10 cells was assessed by confocal microscopy (Spectral Confocal Microscope Zeiss LSM 880). Cells were seeded at a density of 2 × 10^4^ cells onto 20 mm coverslips (previously deposited onto a 24-well plate) and cultured for 24 h. PERFECTA fluorescent emulsion (2 mg/mL) suspended in DMEM or DMEM-F12 for B16-F10 cells and MSCs was, respectively, added to the cells and incubated for 2, 4, 6, 8, and 24 h. Afterward, cells were washed twice with PBS and fixed with 4% paraformaldehyde (PFA) for 30 min. To label the cytoplasmatic actin, cells were finally stained with phalloidin-Alexa488 (Invitrogen), while Draq-5 was used to observe the nuclei. Z-stack orthogonal projections were performed to determine the presence of the droplets inside the cytosol. The cell pellets from B16-F10 cells and MSCs were dissolved in 400 μL of PBS and suspended well before making NMR measurements. For the analysis with DMSO, solid PERFECTA was solved in DMEM or DMEM-F12 with 10% of DMSO in an ultrasonic bath for 1 min. Then, this formulation was incubated with cells for 24 and 48 h at the same doses compared with the PERFECTA lecithin emulsion. Cytotoxicity and ^19^F-NMR measurements on these samples are carried out using the same procedure as those used for the measurement of cell pellets exposed to PERFECTA emulsions.

### EV Isolation and Purification

EVs from control (untreated) and PERFECTA emulsion-treated cells (MSCs and B16-F10) were purified by serial ultracentrifugation cycles.^7^ For the isolation of PERFECTA-EVs^MSCs^ and PERFECTA-EVs^B16-F10^, cells were first incubated with 2 mg/mL of PERFECTA emulsion for 6 h (when the maximum amount of PERFECTA was located within cell cytoplasm). After 6 h, cell cultures were washed three to five times with PBS to eliminate the noninternalized PERFECTA droplets and cells were maintained for 48 h with ULTRACEN media to harvest PERFECTA-EVs (Figure 1). A control experiment for evaluating the possible fraction of PERFECTA emulsion droplets not removed with the washing steps was done by incubating MSCs with PERFECTA emulsion for only 1 min. Then, this sample was subject to the same isolation protocol shown in Figure 1.

To remove the remaining debris, cell supernatants were collected and centrifuged for 20 min at 2000*g* and at 4 °C. For discarding the microvesicles, another centrifugation step was carried out for 30 minutes at 10,000*g* and at 4 °C. Finally, samples were twice ultracentrifuged at 100,000*g* for 2 h at 4 °C to obtain the small extravesicular fraction. EVs^MSCs^ and PERFECTA-EVs^MSCs^ were resuspended in PBS. A Pierce BCA protein assay (Thermo Fisher Scientific) was performed to estimate the protein content in the extravesicular sample. EVs were characterized by an FEI XFEG TITAN electron microscope operated at 300 kV equipped with a CETCOR Cs probe corrector from CEOS Company. Elemental analysis was carried out with an EDS (EDAX) detector, which allows performing EDS experiments in the scanning mode. The electrokinetic potential was estimated by ζ potential measurements at pH = 7 in PBS (Brookhaven 90 plus and ZetaPALS software). PERFECTA association with isolated EVs from MSCs and B16-F10 cells was revealed by ^19^F-NMR measurements. EVs were also characterized by NTA measurements using a NanoSight NS300 system (Malvern Technologies, Malvern, U.K.) configured with a 532 nm laser. The measurements were performed at CNR-SCITEC (CNB group) in Milan. To analyze the expression of control (GAPDH) and extravesicular proteins (CD9, CD81, CD63, and ALIX), Western blotting (WB) was performed.^7^ Briefly, 25 μg of PERFECTA-EVs and control EVs was precipitated with acetone overnight (1:1 v/v), lysated in Laemmli buffer (Sigma-Aldrich), and boiled at 95 °C for 5 min. Proteins were first separated by SDS gel electrophoresis and then transferred to a nitrocellulose membrane at 4 °C. Membranes were blocked overnight with nonfat dry milk in 5% (w/v) of tris-buffered saline (TBS). Before incubating the blots with the antibodies, they were washed with TBS-Tween TBST (three times for 20 min); blots were incubated with primary antibodies GAPDH, 1:1000, CD9; 1:2000 (Abcam U.K.), CD81; 1:500 (Santa Cruz, U.K.), ALIX; 1:1000 (Cell Signaling), and CD63, 1:1000 (BD Biosciences). Membranes were then washed three times with 1% (TBST) followed by incubation of the blots with the secondary antibodies anti-HRP (Sigma-Aldrich). Finally, membranes were again washed with TBST and the chemiluminescence substrate was added and imaging was carried out. The stability of the EVs over time was tested, suspending the EVs in PBS for different time periods (from 1 to 5 days after their purification) at 4 °C. The EVs were precipitated with 1% v/v of acetone and centrifugated. ^19^F-NMR measurements were made on the supernatant to check the presence of a PERFECTA signal. We measured ^19^F-NMR for more than 15 independent EV samples isolated with the standard protocol.

### ^19^F-NMR Properties and MR Imaging Performance of PERFECTA-EVs

^19^F, *T*_1_, and *T*_2_ measurements were performed at 305 K on a spectrometer operating at 400 MHz for the ^19^F nucleus. ^19^F-loading was quantified through an external standard of trifluoroacetic acid (TFA) in D_2_O. ^19^F-NMR spectra were recorded in the range of −65 to −85 ppm compared to TFA (−75 ppm). The NMR probe was maintained at 300 K for the entire duration of the experiment. A delay time between repetitions of 14.5 s was adopted to ensure full relaxation, and 256 scans were collected. An inversion recovery and a CPMG pulse sequence were used for measuring *T*_1_ and *T*_2_, respectively. ^19^F-MRI was performed on samples containing the fluorinated EVs for both cell types. MRI experiments were carried out on a preclinical 7 Tesla MRI scanner (BioSpec, Bruker BioSpin, Ettlingen, Germany) located at the center of experimental imaging, IRCCS San Raffaele Hospital (Milano, Italy). A dual-transmit-receive ^19^F/^1^H volume coil (35 mm × 59 mm) was used for both proton and fluorine MRI. The samples were placed in a falcon tube filled with agarose gel and were imaged at room temperature (21 °C). Both ^1^H and ^19^F-MRI data were acquired using a 3D turbo-spin echo sequence with the same FOV (45 mm × 30 mm × 28 mm) but different matrices (128 × 64 × 8 and 64 × 32 × 8, respectively) and signal averages (8 and 100, respectively). For each sample, the signal-to-noise ratio (SNR) was measured using the image analysis tool of the scanner (Paravision 6.0, Bruker Biospin). The mean signal intensity was measured in the area of each sample on the ^19^F image and divided by the standard deviation of the noise taken in an area of the image outside of the samples. The total amount of 19F atoms was estimated in relation to the SNR of a PERFECTA reference standard (emulsion containing 2 × 10^18 19^F atoms).

### Cellular Viability and Uptake Studies of PERFECTA-EVs

The viability of cells treated with PERFECTA-EVs^MSCs^ and PERFECTA-EVs^B16-F10^ was measured incubating the EVs with both cell lines during 24, 48, and 72 h at 0.044, 0.0875, 0.175, 0.35, 0.7, 1.4, and 2.8 μg/100 μL (expressed as the total extravesicular protein amount). Finally, the PERFECTA-EV uptake was evaluated by confocal microscopy as previously described. MSCs and B16-F10 cells were seeded at a density of 2 × 10^4^ cells onto 20 mm coverslips (in a 24-well plate) and incubated with fluorescent PERFECTA-EVs. Afterward, cells were fixed with 4% PFA and an immunocytochemistry was performed: to label exosome and endosomal pathways, CD63-Alexa488 antibody (Thermo Fisher Scientific) was employed and nuclei were stained with Draq-5. PERFECTA emulsion-based agglomerates were visualized with a 546 nm laser. Z-stack orthogonal projections were developed to determine the presence of PERFECTA aggregates inside the cell cytoplasm.

### In Vivo ^19^F-MRI Tracking Experiments

All of the procedures of this study were performed under the Project Licenses PI 46/20 and AE-biomaGUNE1419 approved by the Ethics Committee for Animal Experiments from the University of Zaragoza (Comisión Ética Asesora para la Experimentación Animal de la Universidad de Zaragoza) and by the CICbioMAGUNE from San Sebastian. Mice were fed ad libitum, and their care and maintenance under specific pathogen-free conditions were carried out according to the Spanish Policy for Animal Protection RD53/2013, which meets the European Union Directive 2010/63 on the protection of animals destined for experimental and other scientific purposes. For these experiments, 6 to 8 weeks old female BALB/c nu/nu mice (Envigo) were employed. All of the animals were maintained under quarantine for 7 days as soon as their arrival at the animal facilities and before starting the experiments. Animal manipulation was carried out under sterile conditions in a hood. For the xenograft model, animals received a subcutaneous injection of 3 × 10^6^ HeLa cells suspended in 200 μL of DPBS. Then, the animals were divided into two different groups of three to four individuals: group A, mice treated with 50 μg of PERFECTA-EVs^MSCs^, and group B, mice treated with an emulsion containing the same amount of PERFECTA. Both animal groups received the same amount of PERFECTA contained in two different formulations (2.51 × 10^19 19^F atoms).

After 15 days from the xenograft tumor inoculation, both formulations were intravenously administered in the tail vein. After 48 h from the administration, mice were imaged by MRI. For MRI, animals were anesthetized and maintained with 2% of isoflurane during the scanner session. Body temperature was maintained at 37 °C using a bed heated with circulating water. MRI experiments were performed using a Bruker MR (Bruker Biospec, Bruker Biospin) with an 11.7 T horizonal bore magnet and a 40 mm volumetric excitation for the detection of ^1^H/19F. Finally, 7 days after the treatment, mice were euthanized by CO_2_ and tumor, kidneys, liver, lungs, spleen, and pancreas were collected for biodistribution and histopathological analysis from the mice treated with PERFECTA-EVs as well as untreated mice. The organs were cut into small pieces with scissors and treated with 2 mL of collagenase solution. Collagenase solution is prepared by dissolving 0.4 mg/mL of collagenase type IV (Sigma cod. C5138) in DMEM (with Ca/Mg), 5% FBS. After incubation at 37 °C to ensure that the organs were digested, the cell suspensions were homogenized, filtered, and washed. Finally, the homogenates were suspended in 400 μL of PBS and ^19^F-NMR was carried out to determine the presence of fluorine in the organs. Organs collected at day 7 were fixed for 24 h with paraformaldehyde (4%, Alfa Aesar) followed by cold 70% ethanol, the organs being finally embedded in paraffin. Three micrometer sections were stained with hematoxylin and eosin (H&E).

### Statistical Analysis

All of the results are expressed as mean ± SD. Statistical analysis of the data and the significant differences among the means were evaluated by two-way analysis of variance (ANOVA) for multiple comparisons by Dunnett’s multiple comparison test (GraphPad Software). Statistically significant differences were indicated as follows: *p < 0.05; **p < 0.01; ***p < 0.0001; and ****p < 0.00001.

## Results and Discussion

### Optimization of PERFECTA Internalization in MSCs and B16-F10 Cells

MSCs and B16-F10-derived EVs were labeled with PERFECTA (PERFECTA-EVs^MSCs^ and PERFECTA-EVs^B16-F10^) leveraging the EV biogenesis pathway. To this end, parental cells were treated with a PERFECTA lipid emulsion (Figure S1) and produced EVs were isolated using an optimized ultracentrifugation protocol (see Experimental Section). We selected MSCs, as there is clear evidence for the preferential migration of MSC-derived EVs to target tumor areas.^7,33−36^ In addition, to verify the reliability and demonstrate the wide potential application of the proposed methodology, a second cell line (melanoma, B16-F10 cells), known to produce EVs in high amount, was also treated with PERFECTA emulsion to produce PERFECTA-EVs^B16-F10^. To set the optimal conditions of incubation for PERFECTA internalization with the selected cell lines, cellular viability studies with the emulsion were performed on both cell lines as a function of PERFECTA concentration (Figure S2A,B). The results showed good cell viability even after 2 days of incubation up to 2 mg/mL for B16-F10 cells, while MSCs displayed more stress upon incubation with PERFECTA emulsion with a viability of around 70–75%. For comparison, PERFECTA was also dissolved in 10% of DMSO and directly added to the cells, but as expected, higher cellular toxicity (Figure S2C,D) and lower PERFECTA internalization were observed (Figure S2E). The cellular uptake of PERFECTA emulsion at different time points was first visualized by confocal microscopy as fluorescent nanodroplets. Confocal microscopy revealed internalization of the fluorescent emulsion in the cytoplasm of both cell lines, with maximum internalization at 6 hours in the case of MSCs and at 24 h for B16-F10 cells (Figure S3A,B). The internalization of PERFECTA by the target cells at 6 h was also confirmed and quantified by ^19^F-NMR measurements (Figure S3C,D). In particular, both cell lines treated with the emulsion showed a sharp and intense signal at a PERFECTA chemical shift (−72.4 ppm), contrarily to control cells. To find the best compromise between the uptake efficiency and cellular viability, 6 h was selected as the optimal incubation time.

### Production and Characterization of Biogenic PERFECTA-EVs as Stable ^19^F-MRI Imaging Tools

**Figure 2:**
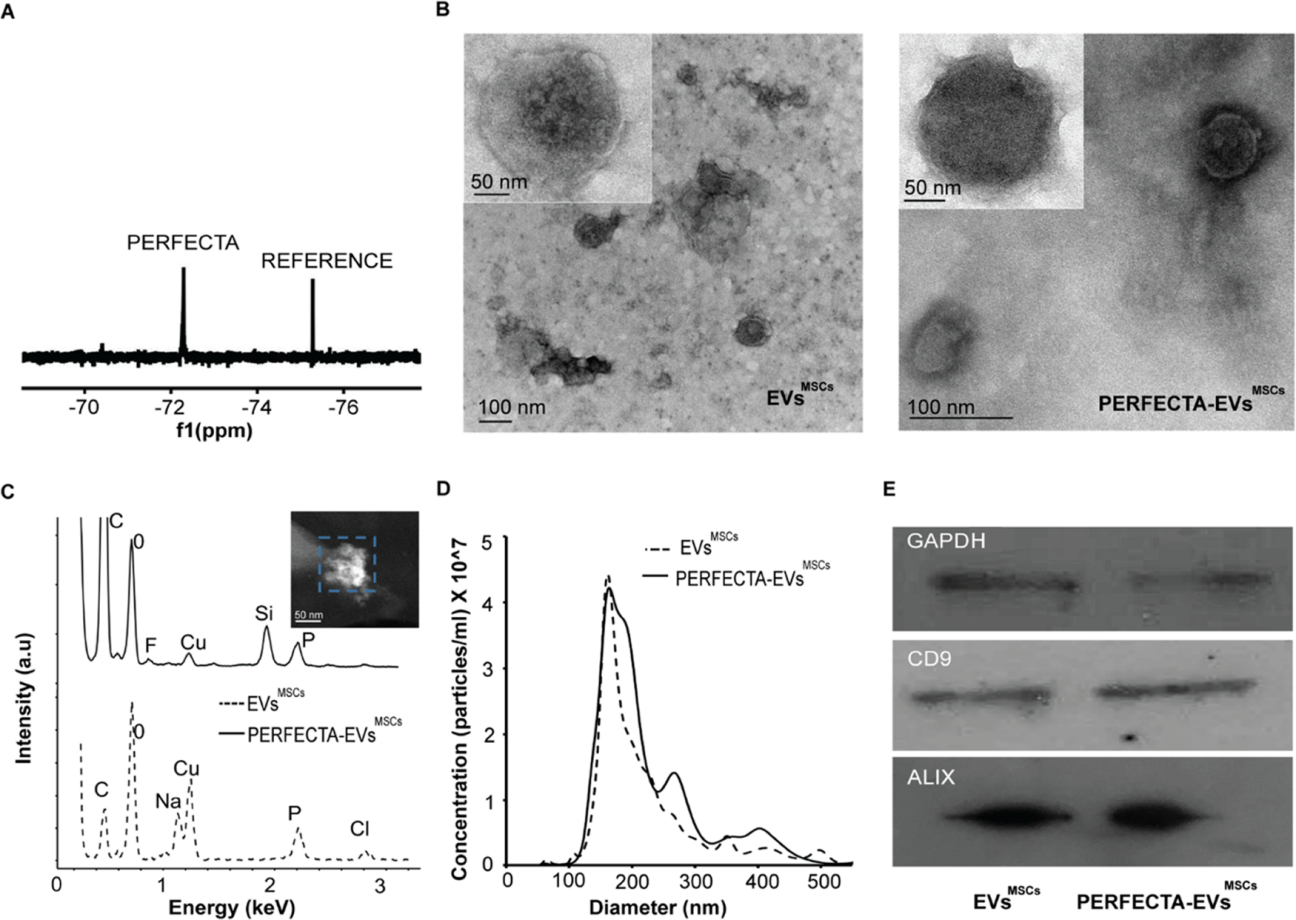
Characterization of biogenic PERFECTA-EVs^MSCs^. (A) ^19^F-NMR spectrum of an aqueous dispersion of isolated PERFECTA-EVs^MSCs^: observed peaks are at −72.44 ppm (PERFECTA) and −75.54 ppm (TFA, external reference). The estimated fluorine content in the dispersion was approximately 1.95 ± 0.9 × 10^8 19^F/EV. (B) Representative TEM images at different magnifications of control and PERFECTA-EVs^MSCs^. (C) EDS–STEM spectrum showing the chemical composition of control and PERFECTA-EVs^MSCs^ with HAADF-STEM image of analyzed PERFECTA-EVs^MSCs^ (inset). The presence of Si in the control sample is a spurious signal due to fluorescence from the supporting grid of the EDS detector window.^39^ (D) EV concentration as n°NPs/mL obtained by the NTA analysis of control and PERFECTA-EVs^MSCs^. (E) WB evaluation of extravesicular (ALIX, CD9) and control (GAPDH) proteins for control and PERFECTA-EVs^MSCS^.

PERFECTA-EVs^MSCs^, PERFECTA-EVs^B16-F10^, and control EVs (EVs^MSCs^ and EVs^B16-F10^) were thoroughly characterized in terms of morphology, size distribution, zeta potential, and specific protein content (Figures 2 and S4). Both aqueous suspensions of labeled and control EVs were first measured by ^19^F-NMR in the presence of an external standard (trifluoroacetic acid, TFA). The spectra of control samples (Figure S4A) exhibited the signal of the reference TFA only, while a sharp signal at the characteristic chemical shift of PERFECTA was clearly observed in the spectra of the labeled EVs from both cell lines (Figures 2A and S4B; see the SI section) corresponding to the presence of PERFECTA in the isolated samples. The control samples obtained incubating MSCs with PERFECTA emulsions for only 1 min showed 10 times lower signal, indicating that possible “free” emulsion droplets not removed by the centrifugation protocol give a negligible ^19^F-NMR signal with respect to the EV-PERFECTA signal (Figure S5). TEM analysis of both PERFECTA-loaded and control EVs revealed lipid vesicles of pseudospherical shape characterized by a diameter of about 100 nm, as previously reported (Figures 2B and S4C);^37,38^ thus, no significant morphological differences were observed between native and PERFECTA-EVs. Importantly, the morphology of the observed EVs is clearly different from that of the initial PERFECTA droplets of the source emulsion (Figure S1A).

Energy-dispersive X-ray spectroscopy (EDS–STEM) demonstrated colocalization of ^19^F atoms with EVs treated with PERFECTA emulsion through the analysis of their chemical composition, while no ^19^F signal was observed associated with untreated EVs (Figure 2C). It is important to note that EDS detection is limited for a low atomic number element (such as fluorine), making the analysis semiquantitative. Nanoparticle tracking analysis (NTA) results showed similar particle size distributions centered at about 170–220 nm for all samples, indicating that both isolated samples are enriched in small-sized EVs with minimal agglomeration, i.e., PERFECTA incorporation in the EVs did not affect their structure and colloidal stability (Figures 2D and S4D and Table 1). Accordingly, ζ potential measurements showed a similar characteristic negative surface charge for both fluorinated and control EVs in both cell lines. Finally, combining EV concentration obtained by NTA measurements with the quantification of PERFECTA content by ^19^F-NMR, it was also possible to qualitatively estimate an average number of ^19^F atoms associated with EV for the two cell lines. A higher PERFECTA loading was found for PERFECTA-EVs^MSCs^, which are meant to be used as possible therapeutic agents. Overall, these results indicated that PERFECTA was associated with cell-derived EVs in a significant amount without compromising their physicochemical features (see Table 1). It was not possible to determine the specific localization of PERFECTA inside the EVs, although given its hydrophobic nature encapsulation seems more likely in the lipid membrane of the extracellular vesicles.

**Table 1:** Physical–Chemical Features of PERFECTA-Loaded MSCs and B16-F10-Derived EVs and Unlabeled EVs

		B16-F10		MSCs	
	PERFECTA emulsion	EVs	PERFECTA-EVs	EVs	PERFECTA-EVs
mean size (nm)^a^	206 ± 7	179 ± 7	193 ± 22	237 ± 81	167 ± 4
ζ potential (mV)	–45 ± 10	– 23 ± 5	– 26 ± 5	–19 ± 7	– 18 ± 6
n° ^19^F atoms/EV^b^	1.63 ± 0.5 × 10^9^		0.88 ± 0.2 × 10^7^		1.95 ± 0.9 × 10^8^

a Mean size was obtained by NTA measurements.

b n° ^19^F atoms/EV were obtained by normalizing the ^19^F atoms measured by ^19^F-NMR with the number of EVs obtained by NTA measurements.

Importantly, EVs^MSCs^ and PERFECTA-EVs^MSCs^ were also characterized by Western blot (WB), evaluating the expression of extravesicular proteins (ALIX and CD63), as well as the presence of a housekeeping protein (GAPDH). WB analysis confirmed that the presence of PERFECTA in the EVs did not affect the expression of either housekeeping or extravesicular proteins (Figure 2E). Moreover, in the case of EVs^B16-F10^ and PERFECTA-EVs^B16-F10^, the expression of neither control proteins (GAPDH) nor tetraspanins specific from EVs (CD9, CD81, and CD63) was affected by the presence of PERFECTA (Figure S4E). Summarizing, characterization results demonstrate that the morphology and integrity of the EVs were maintained upon PERFECTA incorporation. The stability of PERFECTA-EVs in terms of the possible release over time is also shown in Figure S6A,B. Neither PERFECTA-EVs^MSCs^ nor PERFECTA-EVs^B16-F10^ released any detectable amount of PERFECTA in physiological solutions even after 1 week, as expected in a highly hydrophobic molecule. Also, no precipitate/flocculate was observed in the aged dispersions, and ^19^F-NMR experiments of the isolated supernatants, upon EV removal, did not exhibit any PERFECTA signal.

### In Vitro and In Vivo Imaging and Biodistribution of PERFECTA-EVs by ^19^F-MRI

**Figure 3:**
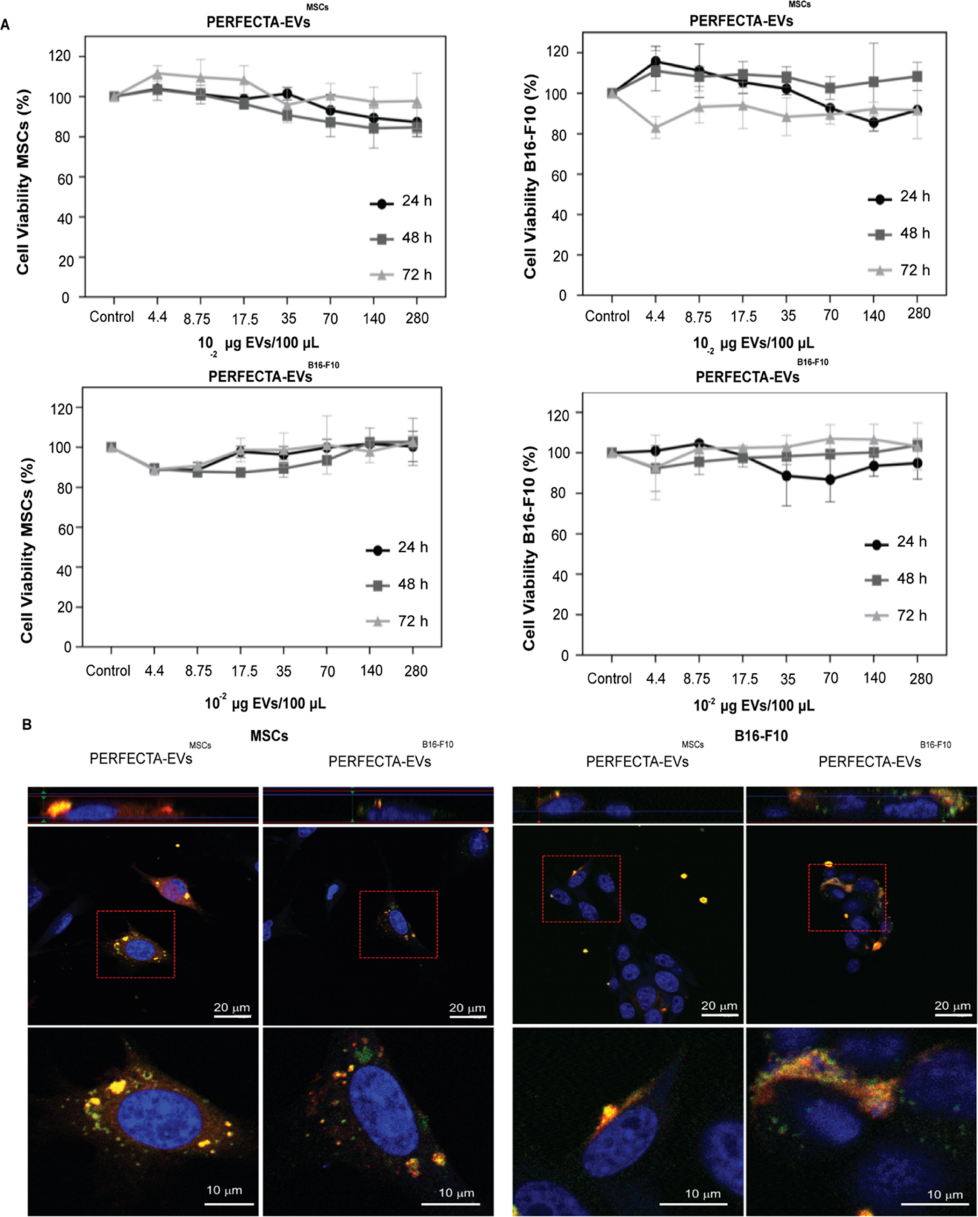
In vitro tests of PERFECTA-EVs^MSCs^. (A) Cytotoxicity evaluation of MSCs and B16-F10 cell lines after 24, 48, and 72 h of incubation with PERFECTA-EVs^MSCs^ (top) and PERFECTA-EVs^B16-F10^ (bottom). (B) Evaluation of PERFECTA-EV^MSCs^ and PERFECTA-EV^B16-F10^ internalization by both cell lines after 6 h of incubation by confocal microscopy: PERFECTA-EVs (red), endosomal and exosomal pathways (green), and nucleus (blue).

The tolerability of MSCs and B16-F10 cells toward exposure to PERFECTA-EVs was also determined by incubating them with increasing amounts of PERFECTA-EVs derived from both cell lines. Viability assays revealed that PERFECTA-EVs did not significantly affect cell viability in either MSCs or B16-F10 cells (Figure 3A), corroborating the biocompatibility of PERFECTA-EVs as imaging tools. Furthermore, PERFECTA-loaded EVs were easily internalized by MSCs and B16-F10 and their presence in cell cytoplasm was confirmed by confocal microscopy. Indeed, cell treatment with a PERFECTA fluorescent emulsion produced fluorescent PERFECTA-EVs. Figure 3B shows fluorescent PERFECTA-EVs as intense red dots in the cell cytoplasm surrounding cell nuclei labeled visualized in blue. In the same image, cellular exosomal and endosomal compartments were labeled using an anti-CD63-Alexa488 antibody and observed in green. Thus, the presence of yellow dots in the cell cytoplasm corresponds to PERFECTA-EVs colocalizing with exosomal and endosomal pathways.

Since the final objective of the study was to enable the visualization of the ability of EVs^MSCs^ to target tumors, the imaging properties such as *T*_1_ and *T*_2_ relaxation times of derived PERFECTA-EVs^MSCs^ were first evaluated in phantoms to assess their suitability for further use in vivo. In fact, there is strong evidence that EVs (i.e., B16-F10) from cancer cells are heavily involved in metastatic tumor growth,^40−42^ which would make their therapeutic use questionable.

**Figure 4:**
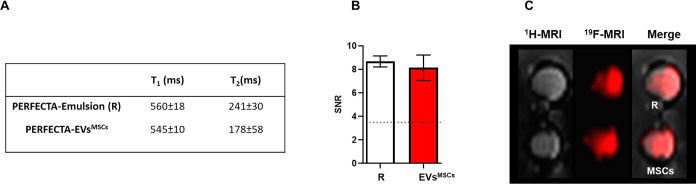
^19^F-MRI properties of biogenic PERFECTA-EVs^MSCs^. (A) *T*_1_ and *T*_2_ relaxation times of the source PERFECTA emulsion (*R*) and isolated PERFECTA-EVs^MSCs^ measured by ^19^F-NMR. (B) Signal-to-noise ratio (SNR) of each sample calculated on ^19^F-MR images. (C) MRI images at ^1^H and ^19^F frequencies were obtained on samples of PERFECTA-EVs^MSCs^ compared with those of PERFECTA emulsion (*R*: 2 × 10^18 19^F atoms). Both ^1^H and ^19^F images were merged for sample identification.

*T*_1_ and *T*_2_ relaxation times for PERFECTA-EV^MSCs^ and PERFECTA-EV^B16-F10^ were determined by ^19^F-NMR experiments (Figures 4A and S7A). Considering that a relatively short *T*_1_ and a long *T*_2_ are usually suitable for imaging purposes,^38,43^ the measured relaxation times for PERFECTA-EV^MSCs^ were promising and comparable to those of the source emulsion as shown in Figure 4A. Accordingly, ^19^F-MRI measurements of phantoms containing suspensions of PERFECTA-EVs^MSCs^ revealed a good signal-to-noise ratio (SNR) (Figures 4B,C and S7B,C) as expected. The obtained results strongly support the possibility of using PERFECTA-EVs for imaging their biodistribution through MRI acquisitions of images with a high SNR using fast imaging sequences.

**Figure 5:**
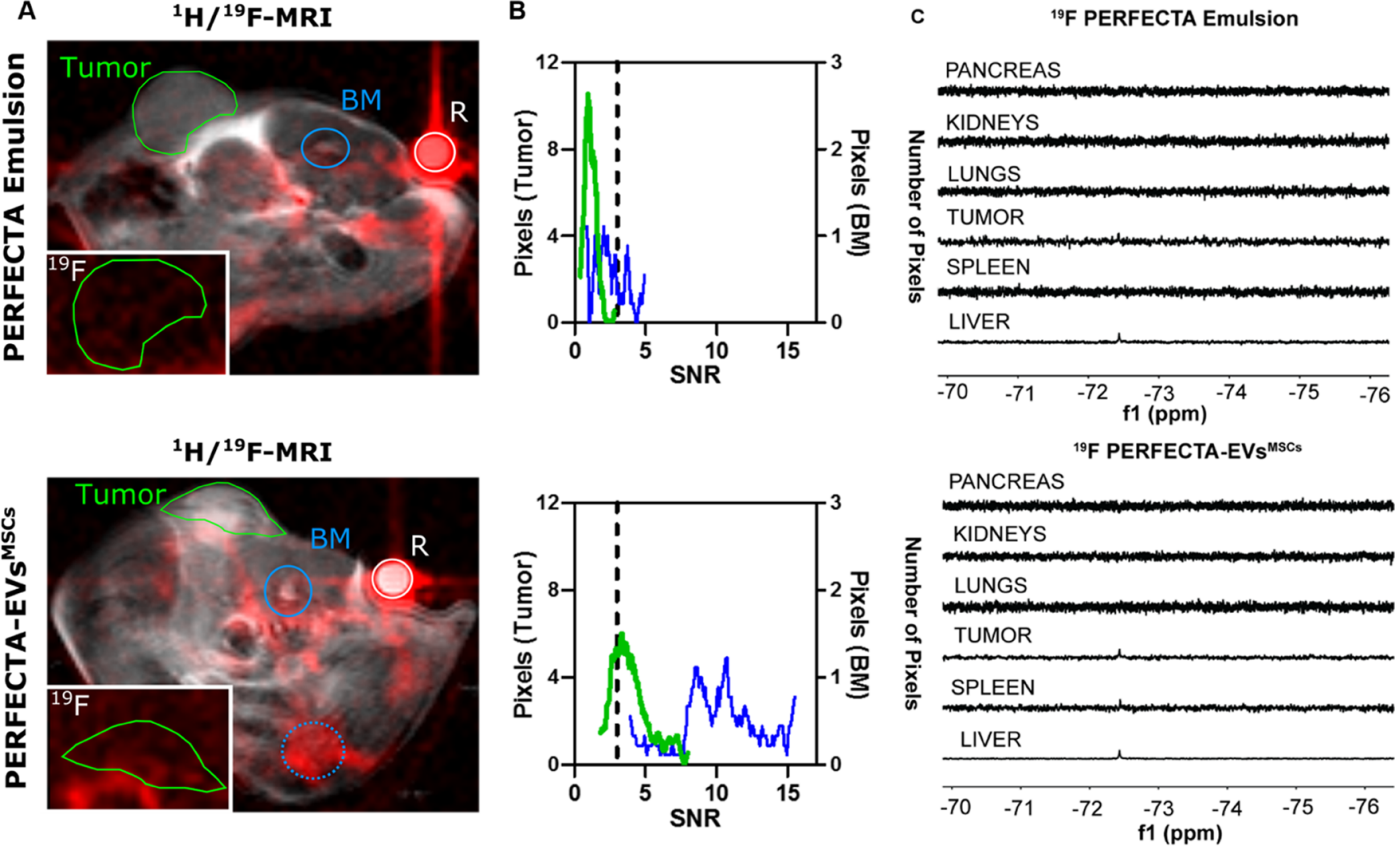
Tumor homing of PERFECTA-EVs^MSCs^. In vivo ^19^F-MRI of BALB-nu mice bearing HeLa tumor: (A) merged ^1^H and ^19^F-MRI (red) images from representative mice after 48 h from the administration of PERFECTA emulsion (top) and PERFECTA-EVs^MSCs^ (bottom). The green circle delineates the tumor region, while the blue circles indicate the bone marrow (BM) of the spinal cord (solid) and the knee (dotted). The white circle in each image indicates the standard reference (*R*). In the white box (bottom-left), ^19^F-MR image magnification focused on the tumor mass. (B) Histograms of ^19^F signal-to-noise ratio (SNR) distribution in both tumor and bone marrow of the spinal cord are reported for each mouse. The dashed line indicates the SNR limit above which ^19^F signal is detectable (SNR > 3). (C) ^19^F-NMR spectra of harvested organs from a representative mouse among those treated with PERFECTA emulsion and those treated with PERFECTA-EVs^MSCs^.

Encouraged by the good imaging properties of PERFECTA-EVs^MSCs^ demonstrated in the experiments with phantoms, experiments with a xenograft tumor-bearing animal model were carried out next. Given the lack of native ^19^F in the animals, the signal obtained would be due exclusively to PERFECTA-EVs^MSCs^ and could be used to confirm their biodistribution and the expected accumulation in the tumor, in view of the known tumor-targeting capabilities of MSC-derived EVs. After 48 h from intravenous administration of 50 μg of PERFECTA-EVs^MSCs^ in the tail of several groups of four mice carrying a xenograft tumor, a fluorine signal from PERFECTA-EVs^MSCs^ was clearly observed mainly in tumor and bone marrow regions (Figure 5A).

On the contrary, in mice treated with PERFECTA source emulsion used as a control, the signal was hardly detectable either in bone marrow or in tumor (Figure 5A,B). Such differences were also confirmed by SNR measurements (Figure 5B), showing that a large portion of tumor exhibited a relevant fluorine signal when the animals received PERFECTA-EVs^MSCs^ compared to control mice (82 vs 31% of the tumor area with a detectable SNR > 3, respectively).

In mice treated with PERFECTA emulsion, the fluorine signal was mainly observed in adipose tissues. The accumulation of the fluorine signal in the bone marrow of mice treated with PERFECTA-EVs^MSCs^ could be related to the nature of the parental cells used to derive the EVs, i.e., precisely stem cells derived from bone marrow. In this regard, targeting of bone marrow could be expected as it has been demonstrated by us and others that EVs retain a fingerprint identity from the cells of origin.^7^ To support these results, ex vivo quantification of the fluorine content in extracted organs from those mice after 1 week of administration of both PERFECTA formulations was performed using ^19^F-NMR (Figure 5C). The isolated organs from mice injected with PERFECTA-EVs^MSCs^ and PERFECTA emulsions were harvested to analyze the content of the fluorinated probe. The harvested organs were homogenized before performing ^19^F-NMR measurements. No fluorine signal from PERFECTA was found in the homogenized pancreas, kidneys, or lungs for both groups of treated mice. On the other hand, a significant signal of PERFECTA was visualized in the liver samples of all mice, while a small ^19^F signal could be observed in the spleen. As observed by ^19^F-MRI, a clear signal was also measured in tumor samples extracted from mice treated with PERFECTA-EVs^MSCs^, whereas no signal was detected in the tumor samples from mice treated with the PERFECTA emulsion. The histopathological analysis of all of the studied organs was carried out on H&E-stained sections. Figure S8 includes representative H&E images of the tumor mass, spleen, liver, and pancreas from control mice and from PERFECTA-EV and emulsion-treated mice at 1 week after treatment. The tumors were histopathologically similar in all of the three cases, despite the intrinsic, unavoidable variation among animals. Some multifocal areas of lymphoplasmacytic infiltration of uncertain interpretation were observed in the peripancreatic fat at 1 week after treatment in both emulsion- and PERFECTA-EV-treated mice. However, in the liver and spleen where most of the PERFECTA signals were seen for treated mice, no morphological alterations that could be attributed to the presence of either EVs or emulsion were observed when compared to control mice.

## Conclusions

Extensive studies performed on EVs have undisputedly established their importance as signal carriers for the intercellular transfer of lipids, proteins, and nucleic acids.^44^ Furthermore, high biocompatibility and preferential migration to selected sites highlight their potential use as promising tools for the detection and treatment of various diseases.^45−50^ The development of novel EV hybrids and tracking protocols would help to accurately and effectively deliver therapeutic and diagnostic agents while providing key information about EV accumulation and distribution in vivo.^7,51^ Herein, we set up an innovative approach to image exogenous EVs by ^19^F-MRI using fluorinated EVs. To produce fluorinated EVs, with intact membranes, their parental cells were incubated with a biocompatible emulsion of the superfluorinated probe PERFECTA, already shown to be internalized by different types of cells.^27,28^ In this way, fluorinated EVs were generated using their natural biogenesis pathway, with no further manipulation. The robustness of the proposed labeling protocol is supported by the fact that labeled EVs could be obtained from different cell lines without significantly altering their viability. Preservation of EV morphology and membrane properties has been shown through multiple measurements comparing PERFECTA-EVs to parental unlabeled EVs. On one side, PERFECTA-EVs maintained excellent ^19^F-NMR characteristics typical of the source emulsion showing a single peak, optimal relaxation times, and stability over time. On the other side, thanks to their intact membranes, they kept the targeting properties exhibited by parental EVs: PERFECTA-EV^MSCs^ accumulation in neoplastic areas was confirmed by in vivo ^19^F-MRI and ex vivo ^19^F-NMR, giving an unequivocal ^19^F signal in the tumor region. Overall, incubation of the target cells with a biocompatible PERFECTA emulsion is a straightforward procedure to obtain PERFECTA-EVs that could be tracked in vivo by ^19^F-MRI. With a highly specific and quantitative MRI signal with no endogenous background, ^19^F-MRI is a superior tool for imaging ^19^F-labeled EVs and can be used to better understand the mechanisms governing the mobility and tropism of EVs. This noninvasive strategy can, in principle, be applied to any type of cell, thus producing a series of parental fluorinated EVs, which could be followed in vivo by ^19^F-MRI. Given the increasing number of EV-based new therapies, PERFECTA-EVs have a strong potential for monitoring the effects of these novel therapeutics, providing real-time information on the status of a particular disease as well as its response to a certain treatment.^52−54^

## Supporting Information Available

Additional results including characterization, cytotoxicity, cellular internalization of PERFECTA emulsions, characterization of PERFECTA-EV^B16-F10s^, stability tests, and H&E images (PDF)

## Abbreviations

EVs: extracellular vesicles
NPs: nanoparticles
NMR: nuclear magnetic resonance
MRI: magnetic resonance imaging
MSCs: mesenchymal stem cells
TEM: transmission electron microscopy
DLS: dynamic light scattering
NTA: nanoparticle tracking analysis
EVs^MSCs^: MSC-derived EVs
EVs^B16-F10^: B16-F10-derived EVs
PERFECTA-EVs^MSCs^: MSC-derived EVs with PERFECTA
PERFECTA-EVS^B16-F10^: B16-F10-derived EVs with PERFECTA
