# Supplementary material for: Superfluorinated Extracellular Vesicles for In Vivo Imaging by 19F-MRI
Source: ACS Appl Mater Interfaces. 2023 Feb 13;15(7):8974–85. doi: 10.1021/acsami.2c20566 (PMC9951174; doi:10.1021/acsami.2c20566)
Supplement: Supplementary file 1 — am2c20566_si_001.pdf [file am2c20566_si_001.pdf]

Supporting Information

Superfluorinated Extracellular Vesicles for *in Vivo* Imaging by ^{19}F-MRI

María Sancho-Albero[1,2,3,‡,†], Nazeeha Ayaz[4,‡], Victor Sebastian[1,2,3], Cristina Chirizzi[4,5], Miguel Encinas-Gimenez[1,2,3], Giulia Neri[4,†], Linda Chaabane[5], *Lluís Luján[6,7], Pilar Martin-Duque[3,8,9,†], Pierangelo Metrangolo[4], Jesús Santamaría[1,2,3*], Francesca Baldelli Bombelli[4,*]*

[1] Instituto de Nanociencia y Materiales de Aragón (INMA), CSIC-Universidad de Zaragoza, 50009 Zaragoza, Spain.

[2] Department of Chemical Engineering and Environmental Technologies, University of Zaragoza, 50009 Zaragoza, Spain.

[3] Networking Research Center on Bioengineering Biomaterials and Nanomedicine (CIBER-BBN), 28029 Madrid, Spain.

[4] Laboratory of Supramolecular and Bio-Nanomaterials (SupraBioNano Lab), Department of Chemistry, Materials and Chemical Engineering, “Giulio Natta”, Politecnico di Milano, 20131 Milan, Italy.

[5] Experimental Neurology (INSPE) and Experimental Imaging Center (CIS), Neuroscience Division, IRCCS Ospedale San Raffaele, 20132 Milan, Italy.

[6] Department of Animal Pathology, University of Zaragoza, 50009 Zaragoza, Spain.

[7] Instituto Universitario de Investigación Mixto Agroalimentario de Aragón (IA2), University of Zaragoza, 50009 Zaragoza, Spain.

[8] Instituto Aragonés de Ciencias de la Salud (IACS) /IIS Aragón, Zaragoza, Spain.

[9] Fundación Araid, 50018 Zaragoza, Spain.

KEYWORDS: Extracellular vesicles, Bioimaging, PERFECTA, ^{19}F-MRI, Fluorine

AUTHOR INFORMATION

* Corresponding authors: Jesus Santamaria (jesus.santamaria@unizar.es) and Francesca Baldelli Bombelli (francesca.baldelli@polimi.it).

† Present addresses:

M. S-A. Department of Molecular Biochemistry and Pharmacology, Instituto di Ricerche Farmacologiche Mario Negri IRCCS, 20156 Milan, Italy.

G.N. Department of Chemical, Biological, Pharmaceutical and Environmental Sciences, University of Messina, 98166 Messina, Italy.

P. M-D. Surgery Department. School of Medicine. University of Zaragoza, 50009 Zaragoza, Spain.

Author Contributions

‡ These authors contributed equally: María Sancho-Albero, Nazeeha Ayaz

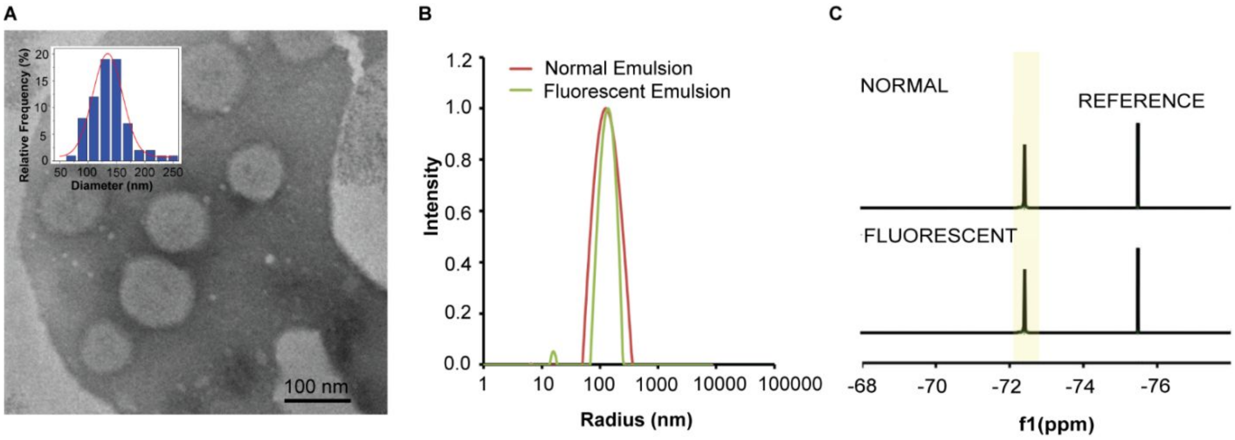

FIGURE S1. A) TEM image showing the morphology of PERFECTA emulsion droplets, the inset reports their size distribution. B) Size analysis by DLS experiments of PERFECTA emulsions showing the size distributions of the fluorescent and unlabelled emulsions at q= 90˚. C) ^{19}F-NMR spectrum of PERFECTA emulsion. The highlighted yellow peak corresponds to PERFECTA at a chemical shift of -72.44 ppm, while the peak at -75.54 ppm is related to TFA, external reference.

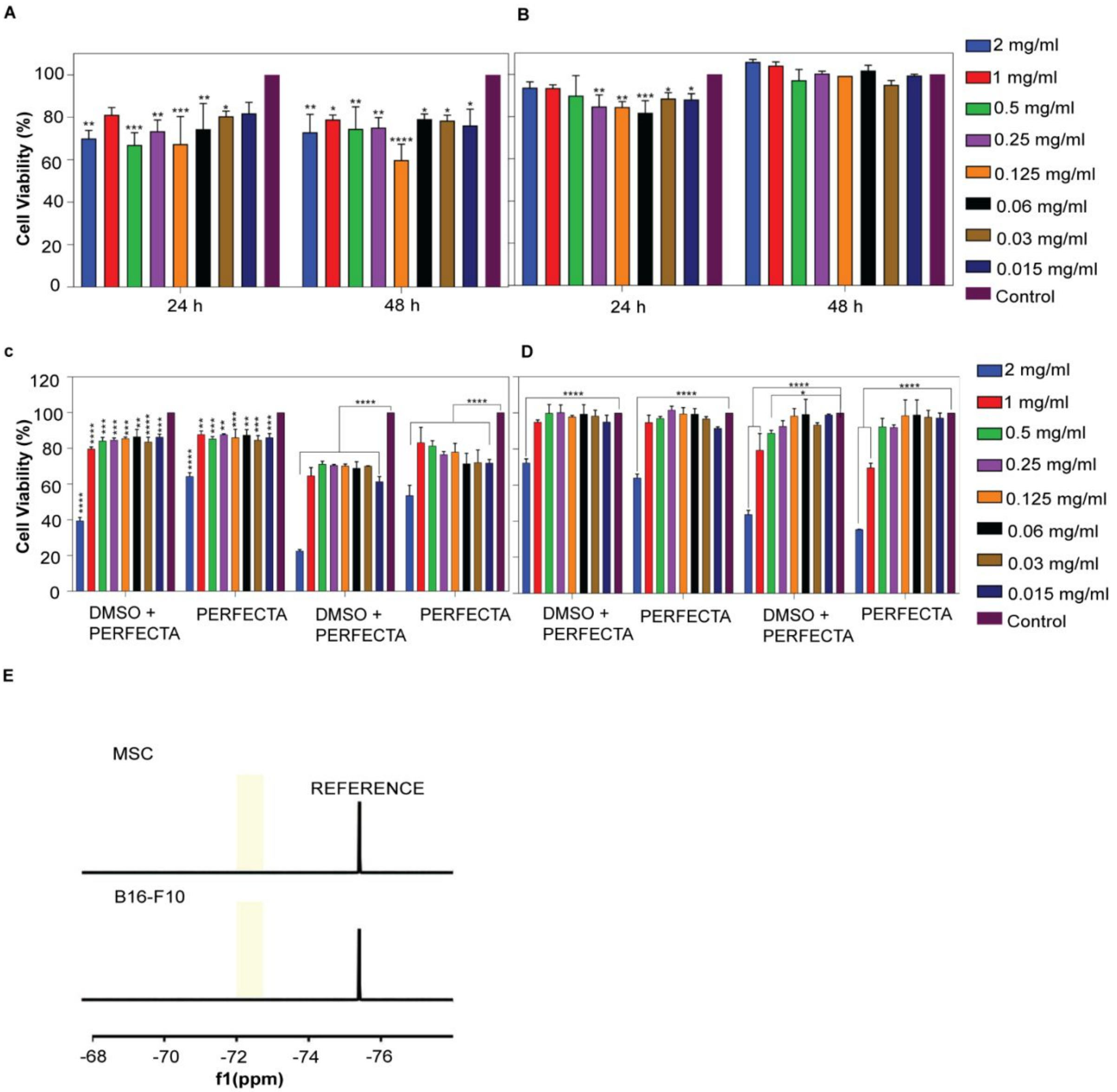

FIGURE S2. Cytotoxicity of (A) MSCs and (B) B16-F10 cells after 24 and 48 h of incubation with PERFECTA emulsion as a function of PERFECTA concentration. Cytotoxicity evaluation of (C) MSCs and (D) B16-F10 cells after 24 and 48 h incubation with PERFECTA-DMSO solution as a function of PERFECTA concentration. (E) ^{19}F-NMR spectra of MSCs and B16-F10 cells incubated with PERFECTA-DMSO solution.

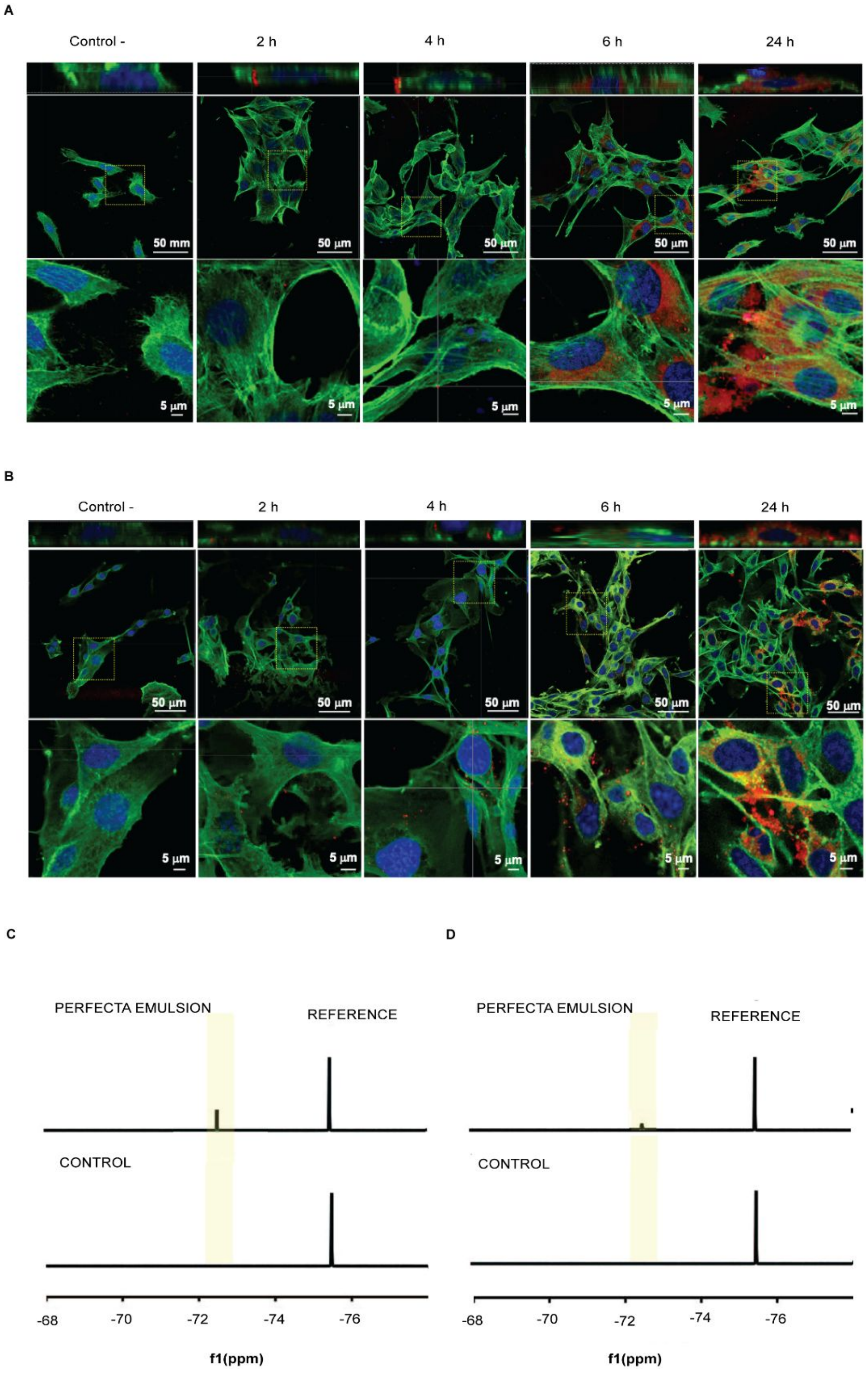

FIGURE S3. Cellular Internalization study by confocal microscopy with PERFECTA fluorescently labelled emulsion at different time of incubation for (A) MSCs and (B) B16-F10 cells. The images show in red the droplet uptake by the cells, in blue the nucleus and in green the

actin. ^{19}F-NMR spectra of (C) MSCs and (D) B16-F10 cells incubated with PERFECTA emulsion for 6 hours and control cells cultured for the same time.

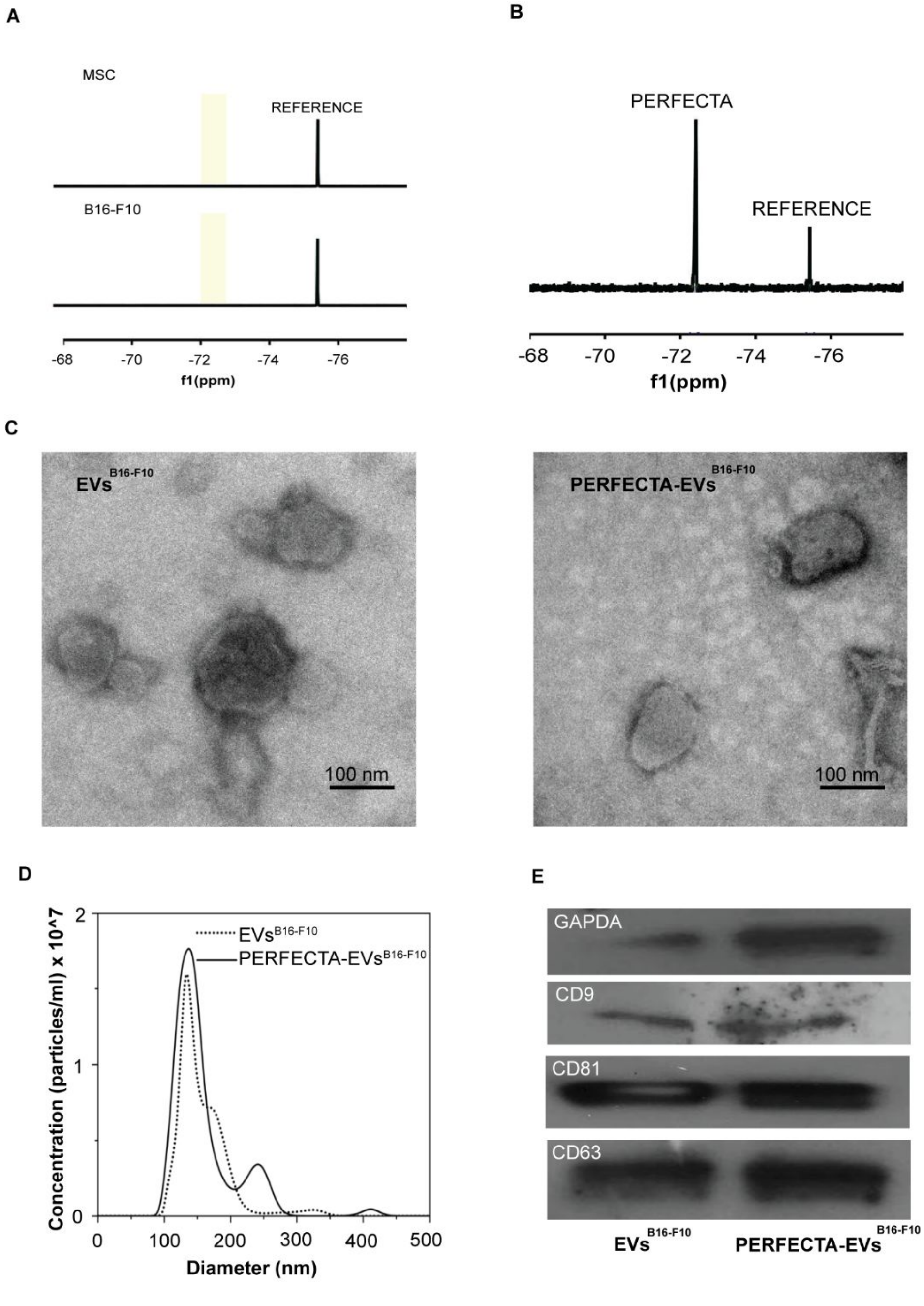

FIGURE S4. Characterization of biogenic PERFECTA-EVs$^{B16\text{-}F10}$. A) ^{19}F-NMR spectra of control EVs from MSC and B16-F10 cell lines showing no PERFECTA signal. (B) ^{19}F-NMR spectrum of an aqueous dispersion of PERFECTA-EVs$^{B16\text{-}F10}$ showing at a chemical shift of -

72.44 ppm the presence of PERFECTA, while the peak at -75.54 ppm is related to TFA, the external reference. Estimated PERFECTA concentration in the dispersion was about 1.4 mM. (C) Representative TEM images of control and PERFECTA-EVs$^{B16\text{-}F10}$. (D) EV concentration as n°NPs/mL obtained by NTA analysis of control and PERFECTA-EVs$^{B16\text{-}F10}$. (E) WB evaluation of extra vesicular (ALIX, CD9) and control (GAPDH) proteins for control and PERFECTA-EVs$^{B16\text{-}F10}$.

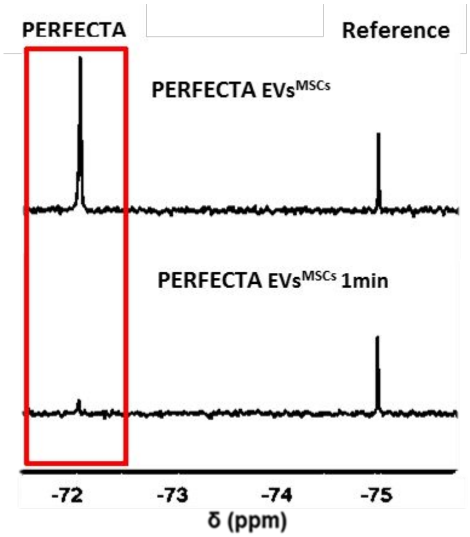

Sample	^{19}F atoms(×10^{18})
PERFECTA-EVs^{MSCs}	1.3
PERFECTA-EVs^{MSCs} 1min	0.13

FIGURE S5. (Left) ^{19}F-NMR of PERFECTA-EV^{MSC} obtained with the conventional protocol and EVs^{MSC} pooled after incubation of the cells with PERFECTA emulsion for 1 minute. (Right) Comparison of PERFECTA-EV^{MSC} and EVs^{MSC} pooled after incubation with PERFECTA for 1 minute.

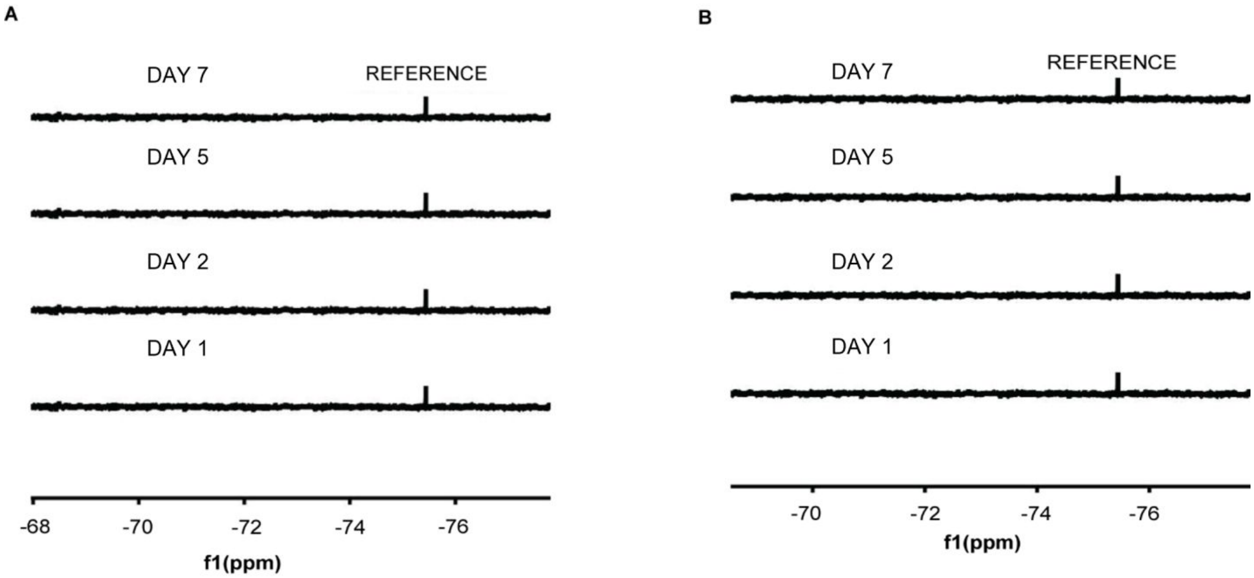

FIGURE S6. ^{19}F-NMR spectra of isolated supernatants from (A) PERFECTA-EVs$^{B16\text{-}F10}$ and (B) PERFECTA-EVs^{MSCs} dispersions incubated in physiological conditions at different times within a week. No PERFECTA signal was detected in these solutions.

A

	T1 (ms)	T2 (ms)
PERFECTA Emulsion	560±18	241±30
PERFECTA-EVs$^{B16\text{-}F10}$	705	308

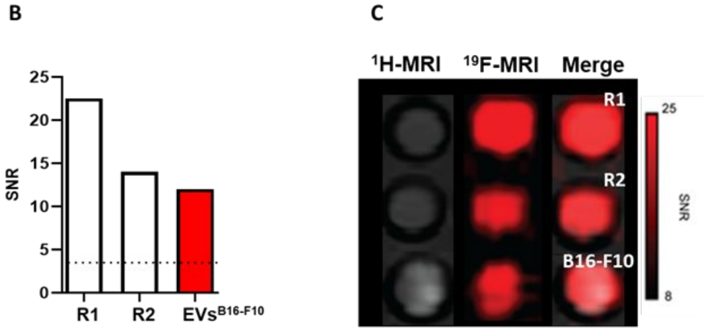

FIGURE S7. ^{19}F-MRI properties of biogenic PERFECTA-EVs$^{B16\text{-}F10}$. A) T_1 and T_2 relaxation times of the source PERFECTA emulsion and isolated PERFECTA-EVs, measured by ^{19}F-NMR. B) Signal to noise ratio (SNR) of each sample calculated on ^{19}F-MR images. C) MRI images at ^{1}H and ^{19}F frequencies were obtained on samples of PERFECTA-EVs$^{B16\text{-}F10}$ compared with PERFECTA emulsion (1 and 2 × 10^{19} F atoms/mL for R1 and R2). Both ^{1}H and ^{19}F images were merged for samples identification

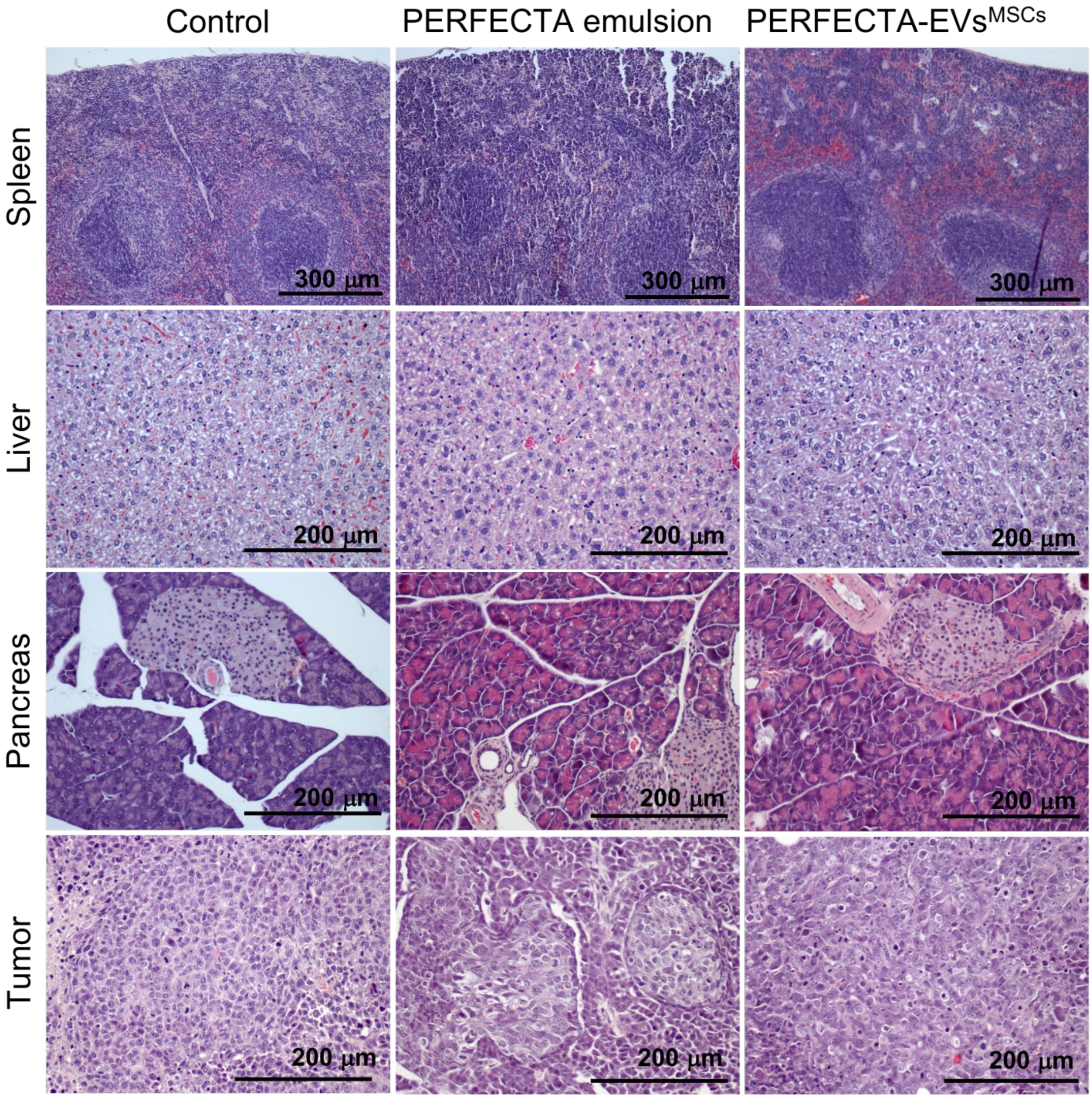

FIGURE S8. Images of spleen, liver pancreas and tumor from control mice (treated with PERFECTA emulsion) and EV treated mice at 1 week after treatment. Bar in first row (spleen): 300 microns. Bar in the rest of images: 200 microns.
